# Rare endocannabinoid system variants identified by whole-exome sequencing and structural modeling in adults with severe obesity predominantly of childhood onset

**DOI:** 10.3389/fendo.2026.1862926

**Published:** 2026-09-25

**Authors:** Kaio Cezar Rodrigues Salum, Ritiele Bastos de Souza, Deborah Antunes, Izadora Sthephanie da Silva Assis, Laura Wendling Gouvêa, Myrela Ribeiro Teixeira, Marília Alves Figueira de Melo, Lohanna Palhinha, Gabriella de Medeiros Abreu, Nathália da Silva Alves, Tamara Silva, Verônica Marques Zembrzuski, Mario Campos Junior, Clarissa Menezes Maya-Monteiro, Patrícia Torres Bozza, Ana Carolina Proença da Fonseca, João Regis Ivar Carneiro

**Affiliations:** 1 Clementino Fraga Filho University Hospital, Federal University of Rio de Janeiro, Rio de Janeiro, Brazil; 2 Molecular Genetics Laboratory, Paulo Niemeyer State Institute of the Brain, Rio de Janeiro, Brazil; 3 Human Genetics Laboratory, Oswaldo Cruz Institute, Oswaldo Cruz Foundation, Rio de Janeiro, Brazil; 4 Laboratory for Applied Genomics and Bioinnovations, Oswaldo Cruz Institute, Oswaldo Cruz Foundation, Rio de Janeiro, Brazil; 5 Laboratory of Immunopharmacology, Oswaldo Cruz Institute, Oswaldo Cruz Foundation, Rio de Janeiro, Brazil; 6 Human Genetics Laboratory, Institute of Biology, Federal Fluminense University, Niterói, Rio de Janeiro, Brazil; 7 Cellular Microbiology Laboratory, Oswaldo Cruz Institute, Oswaldo Cruz Foundation, Rio de Janeiro, Brazil; 8 Genetics Laboratory, Grande Rio University/AFYA, Rio de Janeiro, Brazil

**Keywords:** childhood-onset obesity, endocannabinoid system, FAAH, molecular modeling, rare genetic variants

## Abstract

**Background:**

The endocannabinoid system (ECS) regulates key processes in energy homeostasis, but its contribution to severe obesity beginning in childhood or adolescence remains incompletely understood. This study aimed to identify rare variants in ECS genes and evaluate their potential structural effects.

**Methods:**

A total of 93 adults with severe obesity beginning before 21 years of age (BMI ≥ 35 kg/m²) were recruited from two specialized obesity programs; among participants with onset information, 97.8% reported childhood onset. Whole-exome sequencing was performed, followed by targeted analysis of ECS-related genes. Rare variants (allele frequency <1%) were confirmed by Sanger sequencing and assessed using *in silico* pathogenicity prediction and molecular modeling. *In silico* saturation mutagenesis of all 19 possible substitutions at each variant position was additionally performed using three complementary stability predictors based on different methodological principles (FoldX, Pythia and DDMut), together with AlphaMissense.

**Results:**

Nine rare variants were identified in twelve individuals, seven of which were defined as variants of uncertain significance (VUS). Among the observed variants, *FAAH* p.(Arg326Pro) showed the most concordant predicted impact: it ranked as the most destabilizing substitution at position 326 by FoldX (ΔΔG = +3.04 kcal/mol; rank 1 of 19) and was the only observed variant classified as likely pathogenic by AlphaMissense (0.884). Arg326 lies 24.3 Å from the catalytic triad (Lys142–Ser217–Ser241), consistent with a predicted effect on fold stability rather than direct disruption of the active site. Conversely, at *DAGLA* position 750, all 19 substitutions were predicted as neutral by FoldX and DDMut and as likely benign by AlphaMissense, consistent with predicted structural tolerance at this position. No anthropometric or metabolic parameter differed significantly between carriers and non-carriers after correction for multiple testing.

**Conclusions:**

Rare ECS variants, including a previously unreported *FAAH* p.(Arg326Pro) substitution, were identified in adults with severe obesity, predominantly with childhood onset. These *in silico* analyses are predictive and provide computational evidence for prioritizing candidate variants, notably *FAAH* p.(Arg326Pro); they do not, by themselves, establish pathogenicity or a causal relationship with the phenotype, which will require functional validation.

## 1 Introduction

Obesity is a complex and multifactorial health condition that has escalated into a global public health crisis. Although it is traditionally attributed to lifestyle factors such as poor diet and sedentary behavior, recent advances in biomedical research have revealed the critical role of biological systems in its development (1). One of these systems, the ECS, has gained significant attention due to its role in regulating key processes related to energy balance, appetite, and fat storage (2, 3).

The global prevalence of obesity has surged, affecting an estimated 14% of the world’s population, including 124 million children (4). The growing understanding of the ECS presents new avenues for potential therapeutic interventions aimed at treating this condition (5). The intricate interactions between the ECS and metabolic pathways (lipogenesis, fatty acid oxidation, glucose homeostasis, hypothalamic appetite regulation, and AMPK-mediated energy balance) suggest that targeting this system may provide solutions to obesity, complementing more traditional approaches focused on lifestyle modification (6).

In the past, obesity research primarily concentrated on external factors such as the increasing consumption of ultra-processed foods and the decline in physical activity levels, both of which undoubtedly contribute to the rise in obesity rates (7). However, there is now a broader recognition that obesity is not merely a behavioral or environmental issue but a complex disease influenced by genetic and biological components (8–11). Among these biological factors, the ECS has emerged as a critical regulatory system (5, 6, 12–16).

The ECS consists of cannabinoid receptors (CB1 and CB2) (17–19), endocannabinoids (20–22), and the enzymes responsible for their synthesis and degradation. These components play a pivotal role in various physiological processes, including appetite regulation (23, 24), lipid metabolism, and energy homeostasis (25–27). Recent research has underscored the importance of the ECS in obesity development, especially the role of the CB1 receptor, which is heavily involved in promoting food intake and fat accumulation (6, 15, 28).

The CB1 receptor is predominantly found in the central nervous system, where it influences a range of metabolic and behavioral processes, including hunger, satiety, and energy storage (2, 29). In states of overnutrition, pharmacological inhibition of CB1 receptor activity may lead to a significant reduction in food intake and body weight, demonstrating the therapeutic potential of targeting the ECS for obesity management (30–34). However, the activation of CB1 receptors in adipose tissue promotes lipogenesis, encouraging fat accumulation (35), while the inhibition of these receptors favors lipolysis and the activation of brown adipose tissue, leading to increased energy expenditure (36). This growing body of evidence indicates that obesity cannot be fully understood without considering the biological processes governed by the ECS (3).

Moreover, genetic studies have uncovered important associations between variants in ECS-related genes and obesity. Genetic variation in *FAAH*, which encodes the enzyme responsible for anandamide (AEA) degradation, has been associated with obesity-related traits. The common *FAAH* rs324420 polymorphism has been associated with increased AEA levels (37, 38), lower adiponectin levels (38), higher body mass index (BMI) in various populations (39), and an unhealthy metabolic profile (40, 41). These findings suggest that disruptions in endocannabinoid degradation may contribute to obesity, positioning *FAAH* as a potential genetic marker for the condition.

Similarly, variants in the *CNR1* gene have been associated with childhood and adult obesity in various populations. The c.3813A>G variant was associated with increased subscapular skinfold thickness and waist circumference in Italian and British male individuals (42). Another European study, which included 1,932 obesity cases and 1,173 controls, investigated 26 SNPs in the *CNR1* gene. The intronic variants rs806381 and rs2023239 showed a significant association with both childhood and adult obesity (43). In a study involving 783 male individuals from Denmark, the polymorphisms rs806381 and rs1049353 were investigated and associated with increased visceral fat mass and intermuscular fat, respectively (44).

Additionally, the CB2 receptor, predominantly expressed in immune cells (45–47), has also been implicated in obesity-associated traits (48–52).

The use of next-generation sequencing (NGS) technologies has made it possible to identify rare variants in genes linked to early-onset obesity (53). However, there is a lack of studies examining the impact of rare variants in ECS genes as a potential factor in or cause of human obesity. Investigating these variants could uncover novel molecular pathways involved in obesity, offering targeted opportunities for the development of more refined therapeutic interventions.

## 2 Materials and methods

### 2.1 Patients

This study included 93 adults from Rio de Janeiro, Brazil, with severe obesity beginning before 21 years of age. Inclusion criteria were: BMI ≥ 35 kg/m², obesity onset in childhood (0–11 years) or adolescence (12–21 years), and eligibility for bariatric surgery. Obesity onset was self-reported at the baseline interview as a categorical variable (childhood, 0–11 years; adolescence, 12–21 years); the exact age at onset was not recorded, so participants cannot be stratified by exact age at onset or identified as having onset before five years of age. Exclusion criteria were: (1) pregnancy, (2) lactation, and (3) signs of syndromic obesity, including cognitive delay, dysmorphic features, or organ-specific abnormalities. In addition, before the definition of the final whole-exome sequencing cohort, individuals had been previously screened for MC4R variants by Sanger sequencing as part of earlier molecular studies conducted by our group. Participants carrying pathogenic or likely pathogenic *MC4R* variants were excluded at this pre-WES screening stage and were therefore not included among the 93 individuals analyzed in the present study (54, 55). Participants were volunteers from the Rescue Group for Self-Esteem and Citizenship of the Obese (Grupo de Resgate à Autoestima e Cidadania do Obeso) and the Obesity and Bariatric Surgery Program at Clementino Fraga Filho University Hospital, Federal University of Rio de Janeiro. The study was approved by the Oswaldo Cruz Foundation Ethics Committee (CAAE: 09225113.0.0000/Protocol N°: 346.634), with written informed consent obtained from all participants.

Sociodemographic and anthropometric data were collected during the first interview, including age, sex, weight, height, waist circumference, hip circumference, and neck circumference. BMI and BAI were also calculated.

### 2.2 Whole exome sequencing analysis

Genomic DNA was extracted from peripheral blood samples using the QIAamp Blood Kit (Qiagen, Valencia, CA, USA). DNA concentrations were quantified using a NanoDrop spectrophotometer (Thermo Fisher Scientific, Waltham, MA, USA). Genomic libraries were prepared with the Whole Exome Solution V2 kit (SOPHiA Genetics, Saint-Sulpice, Switzerland), and sequencing was performed on the Illumina NovaSeq 6000 platform (Illumina, San Diego, CA, USA). Bioinformatic analysis was conducted using SOPHiA DDM^®^ software (SOPHiA Genetics, Saint-Sulpice, Switzerland).Whole-exome sequencing achieved a mean on-target depth of 52×, with 99.3% of reads mapped to the reference genome. Overall, 98.9% of targeted bases were covered at ≥20×. A custom virtual panel was designed to screen for rare variants in the *ABHD12*, *ABHD5*, *ABHD6*, *CNR1*, *CNR2*, *DAGLA*, *FAAH*, *GPR55*, *MGLL*, *NAAA*, *NAPEPLD*, and *TRPV1* genes. Rare variants were defined as those with an allele frequency of less than 1% in population databases.

This virtual panel was designed as a hypothesis-driven approach to investigate rare variation in genes involved in the ECS, a pathway with a biologically plausible role in appetite regulation, energy homeostasis, and obesity. The analysis was intended to explore rare ECS-related variants rather than to provide a comprehensive molecular diagnosis of monogenic obesity.

### 2.3 Variant confirmation by sanger sequencing

All nine rare variants identified by whole-exome sequencing were confirmed by bidirectional Sanger sequencing Targeted genomic regions were amplified and sequenced using the ABI 3730xL DNA Analyzer (Applied Biosystems, Foster City, CA, USA) available at the Sequencing Technology Platform RPT01A – IOC/FIOCRUZ. The set of primers were designed using the Primer3Plus software, based on reference sequences retrieved from the Ensembl and NCBI genomic databases. The sequence of primers and PCR conditions is available upon request.

### 2.4 *In silico* analysis

Population frequency databases, including gnomAD (exomes and genomes) (56), ABraOM (57) and dbSNP (58), were queried to determine allele frequencies and apply the predefined rarity criterion (allele frequency <1%). Additional searches in ClinVar (59) and ClinGen (60) were performed to retrieve previously reported clinical interpretations and phenotype associations.

*In silico* analyses were performed using the following pathogenicity prediction tools: AlphaMissense (Google DeepMind, London, UK) (61), SpliceAI (Illumina, Inc., San Diego, CA, USA) (62), and REVEL (Institute for Genomic Medicine, Columbia University, New York, NY, USA) (63). These tools provided comprehensive assessments of variant impact, focusing on missense variants and Splice site disruptions.

Furthermore, the Franklin platform (Genoox Ltd., Palo Alto, CA, USA) (https://franklin.genoox.com - Franklin by Genoox) was utilized for pathogenicity classification, which follows some of the 28 guidelines from the American College of Medical Genetics and Genomics (ACMG). In recent years, ACMG, in collaboration with the Association for Molecular Pathology (AMP) and the College of American Pathologists (CAP), developed detailed guidelines for a more precise interpretation of sequence variants identified in the human genome, driven by the advancement and popularization of next-generation DNA sequencing and other technologies. These guidelines recommend the use of specific standard terminology to describe variants identified in Mendelian diseases, categorizing them as “pathogenic”, “likely pathogenic”, “variant of uncertain significance”, “likely benign”, and “benign” (64).

### 2.5 Molecular modeling analysis of ECS variants

To investigate structural implications of genetic variants in the endocannabinoid system, we conducted comprehensive molecular modeling analyses on key proteins involved in endocannabinoid signaling. The studied proteins were obtained from the UniProt database (65), including Fatty Acid Amide Hydrolase (*FAAH*, O00519), Cannabinoid Receptor 1 (*CNR1*, P21554), N-Acylethanolamine Acid Amidase (*NAAA*, Q02083), Diacylglycerol Lipase Alpha (*DAGLA*, Q9Y4D2), Transient Receptor Potential Cation Channel Subfamily V Member 1 (*TRPV1*, Q8NER1), and N-acyl phosphatidylethanolamine phospholipase D (*NAPEPLD*, Q6IQ20). All residue numbering follows the canonical UniProt sequence and the MANE Select transcript of each gene, and every variant position was verified against the wild-type residue present in the corresponding structure.

Three-dimensional protein structures were obtained through both computational predictions and experimental determinations. For proteins lacking experimental structures, we employed AlphaFold structural predictions (66), specifically for *FAAH* (AF-O00519-F1-v4) and *DAGLA* (AF-Q9Y4D2-F1-v4). Experimental structures were available for *CNR1* (PDB: 9ERX), *NAAA* (PDB: 6DXW), *TRPV1* (PDB: 8GF8), and *NAPEPLD* (PDB: 8P90), which were obtained from the Protein Data Bank (67).

The impact of variants on protein stability was quantitatively assessed using the DDMut deep learning algorithm (68). This analysis provided stability change predictions through ΔΔG calculations, where negative values indicated destabilizing effects and positive values suggested stabilizing effects. To characterize the local structural context of mutations, we analyzed environmental parameters including residue depth, Relative Solvent Accessibility (RSA, a dimensionless ratio), backbone geometry (phi/psi angles), and relative B-factors (69). Structural disorder predictions were performed using the AIUPred2A server (70), which combines energy estimates with deep learning approaches for enhanced accuracy.

*In silico* saturation mutagenesis was performed at each of the eight variant positions, covering all 19 possible substitutions per site (152 in total). Stability effects were predicted with three methods based on different principles: FoldX (empirical force field; five runs per mutation, reported as the mean ± SD) (71), Pythia (self-supervised graph neural network) (72) and DDMut. For FoldX and Pythia, positive values denote destabilization, whereas DDMut uses the opposite sign convention. Pythia returns a score proportional to ΔΔG derived from log-probability ratios and is not calibrated in physical kcal/mol units; it was therefore used for ranking only. Precomputed AlphaMissense scores and classes were retrieved for the same 152 substitutions. The native AlphaMissense categories (likely benign, ambiguous and likely pathogenic) were used for the descriptive and saturation analyses; these categories are distinct from the calibrated ACMG/AMP computational evidence-strength labels (supporting, moderate or strong) reported in **Table 1**. For FoldX, substitutions were classified from the mean ΔΔG (kcal/mol) as stabilizing (ΔΔG < -0.5), neutral (-0.5 ≤ ΔΔG ≤ +0.5), destabilizing (+0.5 < ΔΔG ≤ +2.0) or highly destabilizing (ΔΔG > +2.0); for DDMut, a substitution was counted as neutral when the absolute value of ΔΔG was below 0.5 kcal/mol. For the two predictors computed across the entire protein (Pythia and AlphaMissense), a per-position score was defined as the mean value over the 19 substitutions at that position, and the mutational-constraint percentile of a position was the proportion of all positions in the same protein with a lower mean score. The per-residue confidence of the AlphaFold models was assessed by pLDDT.

**Table 1:** Identified rare variants: allele frequency, functional predictions, and ACMG classification.

Variant information							Allele frequency (*f*)				Clinical significance		Missense variants	Splice altering	Meta scores	Classification according to ACMG
ID	GENE	GRCh38/hg38	Coding consequence	Transcript	cDNA	Protein	dbSNP (RS ID)	gnomAD (exomes)	gnomAD (genomes)	ABraOM	ClinVar	ClinGen	AlphaMissense	SpliceAI	REVEL	Franklin
O85	*FAAH*	1:46408484	missense	NM_001441.3	c.977G>C	p.(Arg326Pro)	Not reported	Not reported	Not reported	Not reported	Not reported	Not reported	Deleterious (Moderate) (0.884)	Benign (0)	Deleterious (Supporting) (0.76)	Uncertain Significance (VUS) (PP3+PM2)
O62	*FAAH*	1:46413472	missense	NM_001441.3	c.1637C>T	p.(Pro546Leu)	rs756925347	0.00002599	0	Not reported	Not reported	Not reported	Uncertain (0.538)	Benign (0)	Deleterious (Moderate) (0.86)	Uncertain Significance (VUS) (PP3+PM2)
O116, O121, O228	*FAAH*	1:46394339	5’UTR	NM_001441.3	c.-10T>G	N/A	rs200388505	0.004750	0.00338	0.008113	Not reported	Not reported	Not reported	Benign (0.05)	Not reported	Likely Benign (PM2+BS2+BP7)
O361	*CNR1*	6:88144596	missense	NM_016083.6	c.679A>G	p.(Ile227Val)	rs1777043051	0.000000684	0.0000131	Not reported	Not Reported	Not reported	Benign (Supporting) (0.211)	Benign (0)	Uncertain (0.32)	Uncertain Significance (VUS) (PM2)
O110, O253	*NAAA*	4:75918784	missense	NM_014435.4	c.975G>T	p.(Leu325Phe)	rs201322204	0.000313	0.00332	0.001708	Not Reported	Not reported	Benign (Supporting) (0.225)	Uncertain (0.17)	Uncertain (0.51)	Uncertain Significance (VUS) (BS2)
O177	*DAGLA*	11:61743609	missense	NM_006133.4	c.2249C>T	p.(Ala750Val)	rs144674730	0.000149	0.00136	0.000427	Likely benign	Not reported	Benign (Moderate) (0.086)	Benign (0)	Benign (Moderate) (0.02)	Likely Benign (PM2+PP2+BP4+BP6+BS2)
O290	*TRPV1*	17:3589991	missense	NM_080704.4	c.860C>T	p.(Thr287Met)	rs200162101	0.0000994	0.000722	Not reported	Not reported	Not reported	Benign (Supporting) (0.161)	Benign (0)	Benign (Supporting) (0.29)	Uncertain Significance (VUS) (PM2+BS2)
O40	*TRPV1*	17:3585838	missense	NM_080704.4	c.1313A>G	p.(Asn438Ser)	rs537774139	0.0000116	0.0000329	Not reported	Not Reported	Not reported	Benign (Moderate) (0.132)	Benign (0.01)	Benign (Supporting) (0.22)	Uncertain Significance (VUS) (PM2)
O224	*NAPEPLD*	7:103120060	missense	NM_001122838.4	c.458G>A	p.(Arg153His)	rs114343410	0.0000937	0.000834	0.000854	Not Reported	Not reported	Benign (Supporting) (0.165)	Benign (0)	Uncertain (0.55)	Uncertain Significance (VUS) (PM2+BS2)

SpliceAI: The score ranges from 0 to 1 and can be interpreted as the probability of the variant being splice-altering; AlphaMissense: It generates scores ranging from 0 to 1, where higher values indicate greater pathogenicity (strong: >0.984, moderate: >0.869, supporting: >0.761) and lower values suggest benign variants (supporting: <0.331, moderate: <0.147, strong: <0.073). Revel: The REVEL score for a missense variant ranges from 0 to 1, where higher values indicate a greater likelihood of the variant being disease-causing. PM2: Exceedingly low frequency in gnomAD databases; BS2: The variant was identified in a homozygous state within population databases at a frequency exceeding what is anticipated for a disease-related mutation; BP7: A synonymous or non-coding variant that is outside of a splice region and is not predicted to have a splice-altering impact; PP3: For a missense variant or one located in a splicing region, computational prediction tools consistently indicate a deleterious impact on the gene; PP2: A missense variant in a gene with a low tolerance for benign missense mutations, where missense changes are a well-established mechanism of disease; BP4: For a missense variant or one located in a splice region, computational prediction tools consistently indicate a benign impact on the gene; BP6: A reputable source has recently classified the variant as benign; however, the supporting evidence is not accessible for independent evaluation by the laboratory (https://franklin.genoox.com - Franklin by Genoox).

Mutations were introduced *in silico* using UCSF ChimeraX’s swapaa command (73), with mutant residue conformations selected using the backbone-dependent Dunbrack rotamer library (74). This library accounts for the influence of local secondary structure on amino acid conformational probabilities, ensuring realistic modeling of variant structures. Structural analysis was performed using UCSF ChimeraX’s molecular visualization capabilities (73), enabling detailed examination of hydrogen bond networks and potential steric clashes resulting from amino acid substitutions.

### 2.6 Statistical analysis

Statistical analyses were performed in Python 3 using the SciPy library (75). Continuous variables are summarized as median (first; third quartile), computed with the exclusive quartile definition. Given the small number of variant carriers and without assuming normality of the clinical variables, carriers of rare ECS variants were compared with non-carriers using the two-sided Mann-Whitney U test (76), a distribution-free method, across sixteen anthropometric and metabolic parameters: age, weight, BMI, waist, hip and neck circumference, waist-to-hip ratio, total cholesterol, LDL, HDL, triglycerides, fasting glucose, HbA1c, C-reactive protein, leptin and adiponectin. To limit false-positive findings arising from multiple comparisons, p-values were adjusted with the Benjamini-Hochberg procedure at a false discovery rate of 5% (77). Missing values were not imputed; each parameter was analyzed using all participants with an available measurement, and the group sizes are reported in **Supplementary Table S2**. The 95% confidence interval for the carrier frequency was estimated with the Wilson score method (78), which is more accurate than the normal approximation for small samples and for proportions away from 0.5. In line with the descriptive, hypothesis-generating design of the study, no *a priori* power calculation was performed and the analysis was not intended to test for statistical enrichment of variants; this is acknowledged in the Discussion.

## 3 Results

### 3.1 Phenotypic characterization of the study population

A total of 93 individuals were enrolled in this study, with a median BMI of 48.9 (45.4; 56.4). The median age was 39 years (29.0; 47.0), the median weight was 134.0 kg (118.0; 155.0), and the median height was 1.63 m (1.57; 1.70). The median waist circumference was 138.0 cm (128.5; 147.5), and the median hip circumference was 146.0 cm (136.0; 156.0), resulting in a median waist-to-hip ratio of 0.97 (0.90; 1.01). Among the participants, 78% were female and 22% were male.

Obesity onset was recorded for 91 of the participants. Of these, 89 (97.8%) reported onset during childhood and two reported onset during adolescence; all twelve carriers of rare ECS variants reported childhood onset. The cohort is therefore predominantly, though not exclusively, childhood-onset. Carriers and non-carriers did not differ significantly for any of the sixteen anthropometric and metabolic parameters examined after correction for multiple testing (**Supplementary Table S2**). Two parameters reached nominal significance before correction, hip circumference (p = 0.047) and adiponectin (p = 0.047), and neither remained significant after the Benjamini-Hochberg procedure (q = 0.32 for both). The frequency of carriers was 12 of 93 (12.9%; 95% CI 7.5-21.2%).

Our analysis identified a total of nine rare variants distributed among twelve individuals. Three variants were located in the *FAAH* gene: c.977G>C; p.(Arg326Pro), c.1637C>T; p.(Pro546Leu), and a 5’ untranslated region (5’UTR) variant, c.-10T>G. Additional rare variants were detected in the *CNR1* c.679A>G; p.(Ile227Val), *NAAA* c.975G>T; p.(Leu325Phe), *DAGLA* c.2249C>T; p.(Ala750Val), *TRPV1* (two variants: c.860C>T; p.(Thr287Met) and c.1313A>G; p.(Asn438Ser)), and *NAPEPLD* c.458G>A; p.(Arg153His) genes. Most of the identified variants were classified as missense, except one variant located in the 5’UTR of *FAAH*. Notably, some variants, including *FAAH* c.977G>C p.(Arg326Pro), had not been previously reported in population databases. The anthropometric and metabolic characteristics of the individuals harboring these variants are summarized in **Table 2**.

**Table 2:** Anthropometric and metabolic characteristics of individuals with rare variants.

Variables	n	Values
Age (years)	12	42.5 (34.5; 48)
Sex		
Female	9	75%
Male	3	25%
Weight (kg)	12	123.5 (104.1; 145.1)
Height (m)	12	1.62 (1.53; 1.69)
BMI (kg/m²)	12	46.4 (44.5; 49.9)
BAI (%)	12	49.2 (44.3; 54.1)
Waist circumference (cm)	12	136.3 (126.3; 143.5)
Hip circumference (cm)	12	137.0 (133.0; 147.9)
WHR	12	0.98 (0.91; 1.01)
Neck circumference (cm)	12	42.8 (40.0; 45.9)
Total cholesterol (mg/dL)	11	198.0 (153.0; 227.0)
HDL cholesterol (mg/dL)	11	43.0 (40.0; 52.0)
LDL cholesterol (mg/dL)	10	122.7 (96.0; 151.7)
VLDL cholesterol (mg/dL)	10	23.0 (19.8; 35.3)
Triglycerides (mg/dL)	11	121.0 (105.0; 179.0)
HbA1c (%)	9	6.2 (4.9; 6.7)
SBP (mmHg)	7	128.0 (126.0; 177.0)
DBP (mmHg)	7	90.0 (81.0; 106.0)

Data are presented as median (25th percentile; 75th percentile) for continuous variables, and as number (percentage) for categorical variables. BAI, Body Adiposity Index; BMI, Body Mass Index; HbA1c, Glycated Hemoglobin; HDL, High-Density Lipoprotein cholesterol; LDL, Low-Density Lipoprotein cholesterol; VLDL, Very-Low-Density Lipoprotein cholesterol; SBP, Systolic Blood Pressure; DBP, Diastolic Blood Pressure; WHR, Waist-to-Hip Ratio.

### 3.2 *In silico* analysis

*In silico* analysis of missense variants in genes involved in the endocannabinoid system revealed heterogeneous pathogenicity profiles. Different *in silico* prediction algorithms, including AlphaMissense, SpliceAI, and REVEL, were employed to stratify variants. AlphaMissense scores ranged from 0.086 to 0.884 among the eight missense variants; the highest score, observed for *FAAH* p.(Arg326Pro), was classified as likely pathogenic, while the remaining variants showed lower scores. SpliceAI predominantly yielded very low predicted splice-altering scores (0-0.01), and REVEL scores suggested varying levels of potential pathogenicity. Most identified variants (**Table 1**) were classified as Variants of Uncertain Significance (VUS) according to the automated ACMG criteria generated by the Franklin tool, which provides a standardized preliminary interpretation in the absence of complete clinical, familial, or functional data required for full manual classification. All identified variants met the predefined rarity criterion of an allele frequency below 1% in the population databases evaluated. The discordant predictions across different *in silico* algorithms emphasize the challenges in accurately predicting the functional consequences of missense variants.

### 3.3 Structural and saturation analysis of the variant positions

Molecular modeling of ECS variants revealed distinct patterns of structural perturbation. Each position was additionally characterized by saturation analysis, in which all 19 possible substitutions were evaluated (**Supplementary Table S1**, **Supplementary Figure S1**). For the two proteins modeled by AlphaFold, per-residue confidence at the variant positions differed markedly: the *FAAH* model had a mean pLDDT of 95.4 and very high local confidence at both sites (98.9 at Arg326 and 98.7 at Pro546), whereas the *DAGLA* model had a mean pLDDT of 63.0 and low confidence in the region of Ala750.

The *FAAH* Arg326Pro variant showed the strongest and most concordant predicted impact, supported by both structural analysis (DDMut ΔΔG = -1.11 kcal/mol) and a high AlphaMissense pathogenicity score (0.884) (**Figure 1A**). Located in a partially exposed region (RSA = 0.34), the arginine side chain forms modeled hydrogen bonds with the backbone of Leu465 and Asp466 (**Figure 1B**). The introduction of proline removes the side chain that donates these hydrogen bonds and, in the modeled rotamer, produces a steric overlap with Asp468 (**Figure 1C**). Arg326 also forms a salt bridge with Asp466 (NE–OD2, 3.78 Å). The residue lies 24.3 Å from the nearest catalytic residue (Ser241), well outside the active site of the enzyme (catalytic triad Lys142–Ser217–Ser241); the predicted structural effect is therefore unlikely to involve direct perturbation of the active site and may instead reflect altered fold stability. Saturation analysis refined this interpretation: position 326 is only moderately constrained (AlphaMissense classifies 7 of 19 substitutions as likely benign, 5 as ambiguous and 7 as likely pathogenic), yet proline is the most destabilizing substitution at this site by FoldX (ΔΔG = +3.04 kcal/mol, rank 1 of 19). Among the eight observed missense variants, p.(Arg326Pro) was the only one classified as likely pathogenic by AlphaMissense. This pattern is consistent with a substitution-specific effect of proline, potentially related to its conformational constraints on the protein backbone, rather than with a uniform intolerance of position 326.

**Figure 1:**
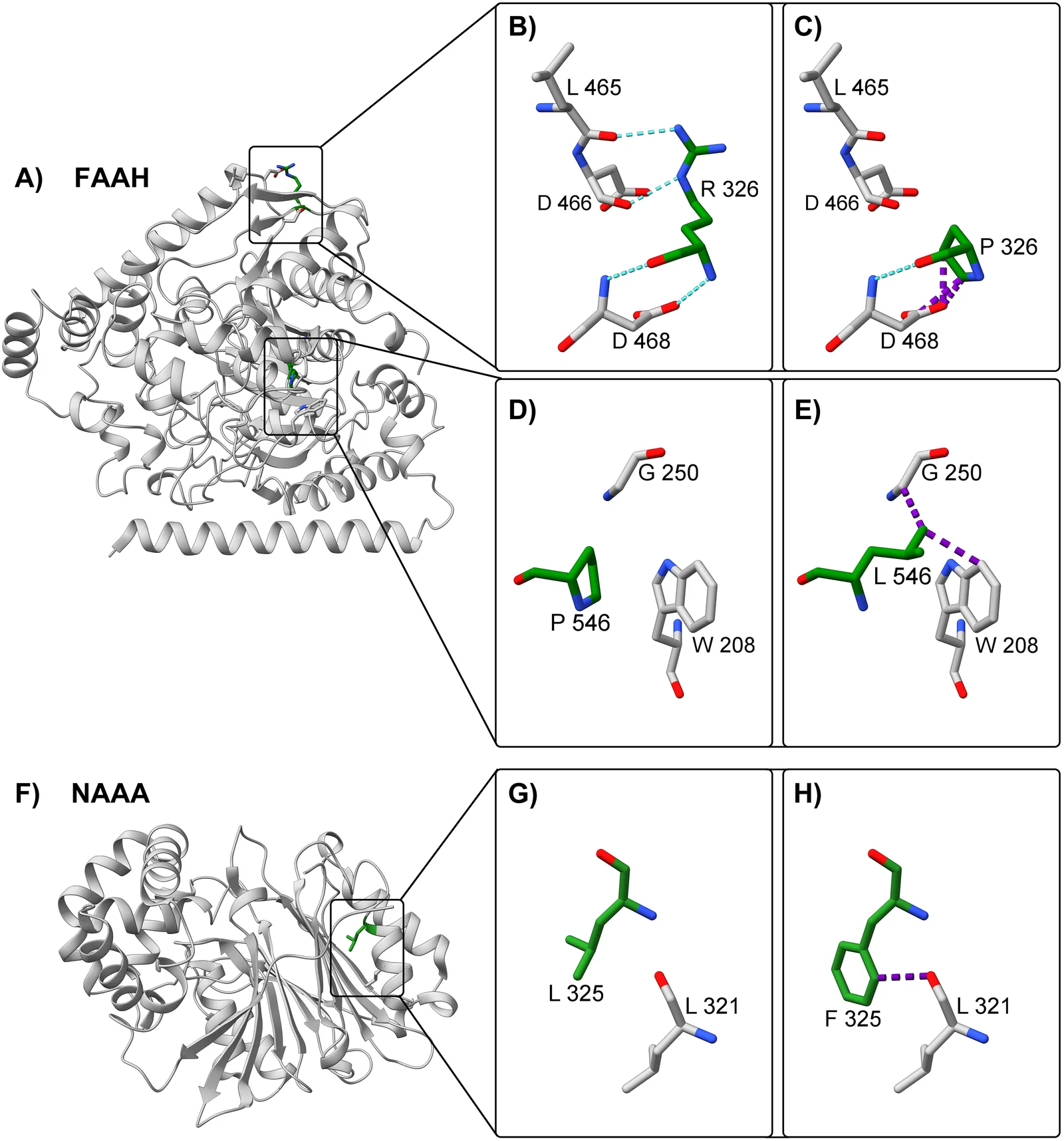
Structural characterization of the *FAAH* and *NAAA* variants. **(A)** Overall structure of *FAAH* with mutated residues highlighted in green. **(B)** Wild-type Arg326 forms hydrogen bonds (blue dashed lines) with Leu465 and Asp466 backbones and a salt bridge with the Asp466 side chain. **(C)** Arg326Pro substitution removes these modeled interactions and produces a modeled steric overlap with Asp468. **(D)** Wild-type Pro546 in native conformation. **(E)** The Pro546Leu substitution introduces steric clashes with Gly250 and Trp208. **(F)** Overall structure of *NAAA* with Leu325 highlighted in green. **(G)** Wild-type Leu325 in native conformation. **(H)** The Leu325Phe substitution creates a steric clash with the Leu321 backbone. Blue dashed lines represent hydrogen bonds, and purple dashed lines indicate steric overlaps between the modeled rotamer and the surrounding residues. D, Asp; F, Phe; G, Gly; L, Leu; P, Pro; R, Arg; W, Trp.

A second *FAAH* variant, Pro546Leu, exhibited notable structural destabilization (DDMut ΔΔG = -1.68 kcal/mol) despite an intermediate AlphaMissense classification (score 0.538). Its location in a highly buried position (RSA = 0.04) and a modeled steric overlap with Gly250 and Trp208 suggest potential consequences for local packing (**Figures 1D, E**). Position 546 is one of the most constrained sites in *FAAH*: all 19 substitutions were highly destabilizing by FoldX (median ΔΔG = +3.94 kcal/mol) and 15 of 19 were classified as likely pathogenic by AlphaMissense. Within this fragile site, however, leucine ranks among the better tolerated options (FoldX rank 14 of 19; AlphaMissense 0.537, ambiguous), so the absolute ΔΔG overstates the impact expected of this particular substitution.

The *NAAA* Leu325Phe variant showed discordant predictions, with severe predicted structural destabilization (DDMut ΔΔG = -2.16 kcal/mol) but a low AlphaMissense pathogenicity score (0.225) (**Figure 1F**). Its completely buried position (RSA = 0.0) and modeled steric overlap with the Leu321 backbone indicate potential effects on local protein packing (**Figures 1G, H**). Leu325 lies 7.2 Å from position 126, the catalytic cysteine generated by autoproteolytic activation of *NAAA*, and contacts Thr127 and Ile129 immediately adjacent to it; the residue therefore occupies a second shell around the active site. Saturation analysis places the substitution in context: 17 of 19 substitutions at Leu325 are highly destabilizing by FoldX (median ΔΔG = +4.48 kcal/mol), whereas phenylalanine is among the mildest (rank 17 of 19) and is classified as likely benign by AlphaMissense. The two stability predictors did not agree at this position (Spearman rho = -0.08 across the 19 substitutions), and the discordance could not be resolved.

The remaining variants showed lower predicted structural impact, although not always concordantly across methods. The *CNR1* Ile227Val variant showed minimal structural destabilization (DDMut ΔΔG = -0.22 kcal/mol) and a low AlphaMissense score (0.211), consistent with its conservative nature (**Figure 2A**). Located in a transmembrane domain, this isoleucine to valine substitution maintains hydrophobic packing at a moderate residue depth (2.01 Å) (**Figures 2B, C**), indicating that the local hydrophobic environment is preserved in the model. Although position 227 is moderately constrained (15 of 19 substitutions likely pathogenic by AlphaMissense), valine is the mildest of the 19 by AlphaMissense and is classified as neutral by FoldX (ΔΔG = +0.49 kcal/mol). The *NAPEPLD* Arg153His variant (**Figure 2D**), positioned at the homodimer interface, introduces a modeled steric overlap with Lys163 of the same chain (**Figures 2E, F**) but shows only modest destabilization (DDMut ΔΔG = -0.20 kcal/mol) and low predicted pathogenicity (score 0.165). Histidine retains a titratable, partially charged side chain at this position, which may explain the mild prediction by DDMut and AlphaMissense. The methods disagreed for this variant: FoldX ranked histidine as the most destabilizing substitution at position 153 (ΔΔG = +3.31 kcal/mol, rank 1 of 19), consistent with alteration of a buried interchain contact at the dimer interface (Arg153 of chain A lies 3.07 Å from Asn194 of chain B), whereas DDMut classified it as essentially neutral and AlphaMissense as the mildest of the 19.

**Figure 2:**
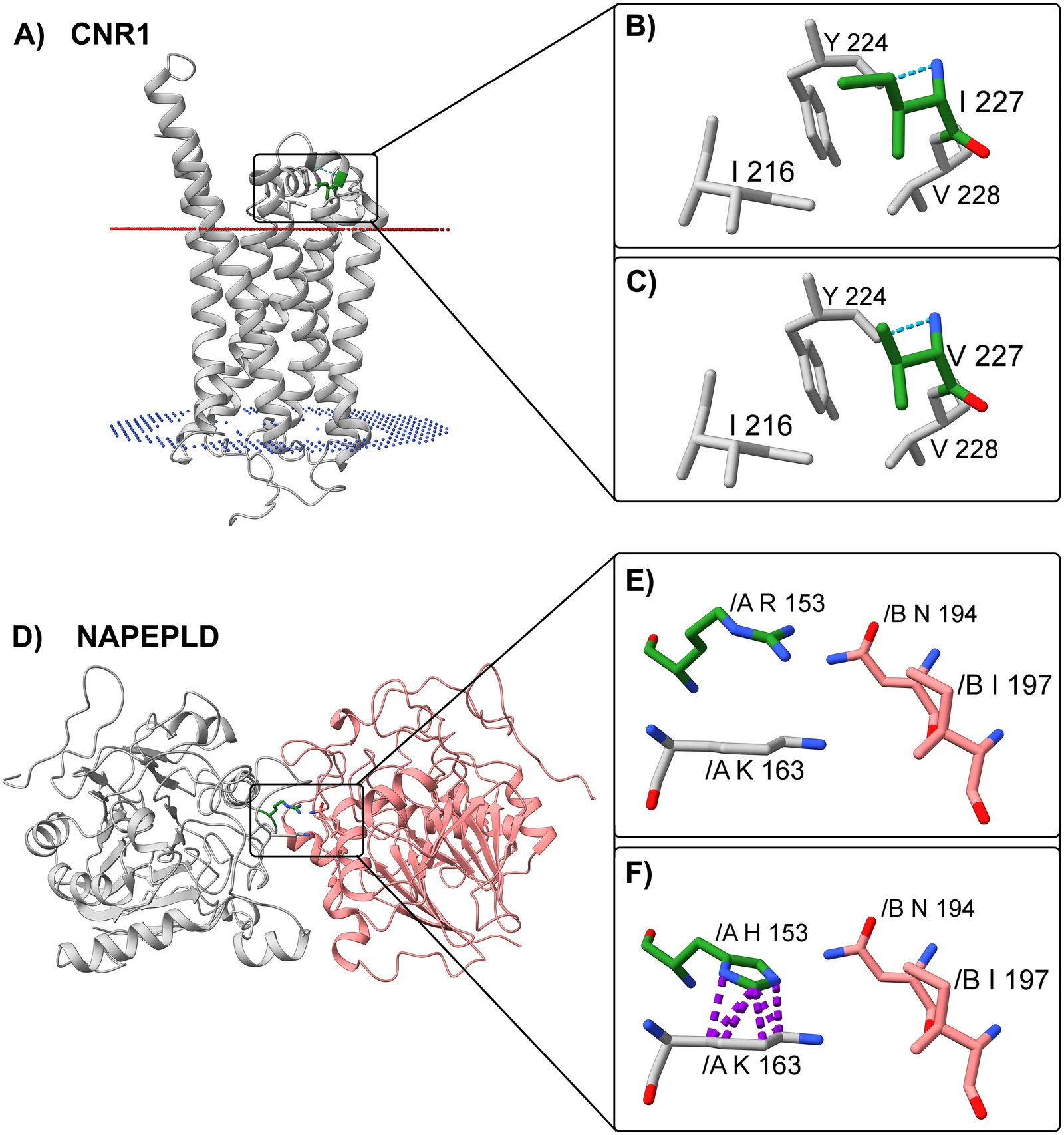
Structural characterization of the *CNR1* and *NAPEPLD* variants. **(A)** Overall structure of *CNR1* (chain R of PDB 9ERX) showing its transmembrane topology with the plasma membrane indicated by red (extracellular) and blue (intracellular) lines. **(B)** Wild-type Ile227 forms hydrophobic interactions with surrounding residues Tyr224, Ile216, and Val228. **(C)** Ile227Val substitution maintains similar modeled hydrophobic contacts. **(D)** Overall structure of *NAPEPLD* homodimer with chains A (gray) and B (salmon). **(E)** Wild-type Arg153 at the dimer interface. **(F)** Arg153His substitution introduces a steric overlap with Lys163 of the same chain, indicated by purple dashed lines. H, His; I, Ile; K, Lys; N, Asn; R, Arg; V, Val; Y, Tyr.

*TRPV1* harbored two variants (Thr287Met and Asn438Ser) with negligible structural impacts (DDMut ΔΔG = -0.26 and 0.07 kcal/mol, respectively) and low pathogenicity scores (0.161 and 0.132) (**Figure 3A**). Thr287Met disrupts a native hydrogen bond with His290 and produces modest steric overlaps with Ser279 and Ala280 in the modeled conformation (**Figures 3B, C**), while the modeled Asn438Ser substitution retains a hydrogen bond with the Ile434 backbone (**Figures 3D, E**). The surface-exposed position of Asn438Ser (RSA = 0.35) is consistent with the predicted structural tolerance of this position. Saturation analysis distinguished the two sites. Position 287 is constrained (17 of 19 substitutions destabilizing or highly destabilizing by FoldX), yet methionine is classified as likely benign by AlphaMissense. Position 438 is tolerant: 14 of 19 substitutions were neutral or stabilizing by FoldX (median ΔΔG = -0.06 kcal/mol), 15 of 19 were neutral by DDMut (median ΔΔG = +0.02 kcal/mol), and the position ranks in the 39th percentile of mutational sensitivity across *TRPV1* by Pythia. By DDMut, both observed substitutions ranked among the mildest at their positions (Thr287Met, rank 16 of 19; Asn438Ser, rank 15 of 19). AlphaMissense, however, classifies 14 of 19 substitutions at position 438 as likely pathogenic, so tolerance at this site is supported by the stability predictors but not by the pathogenicity predictor.

**Figure 3:**
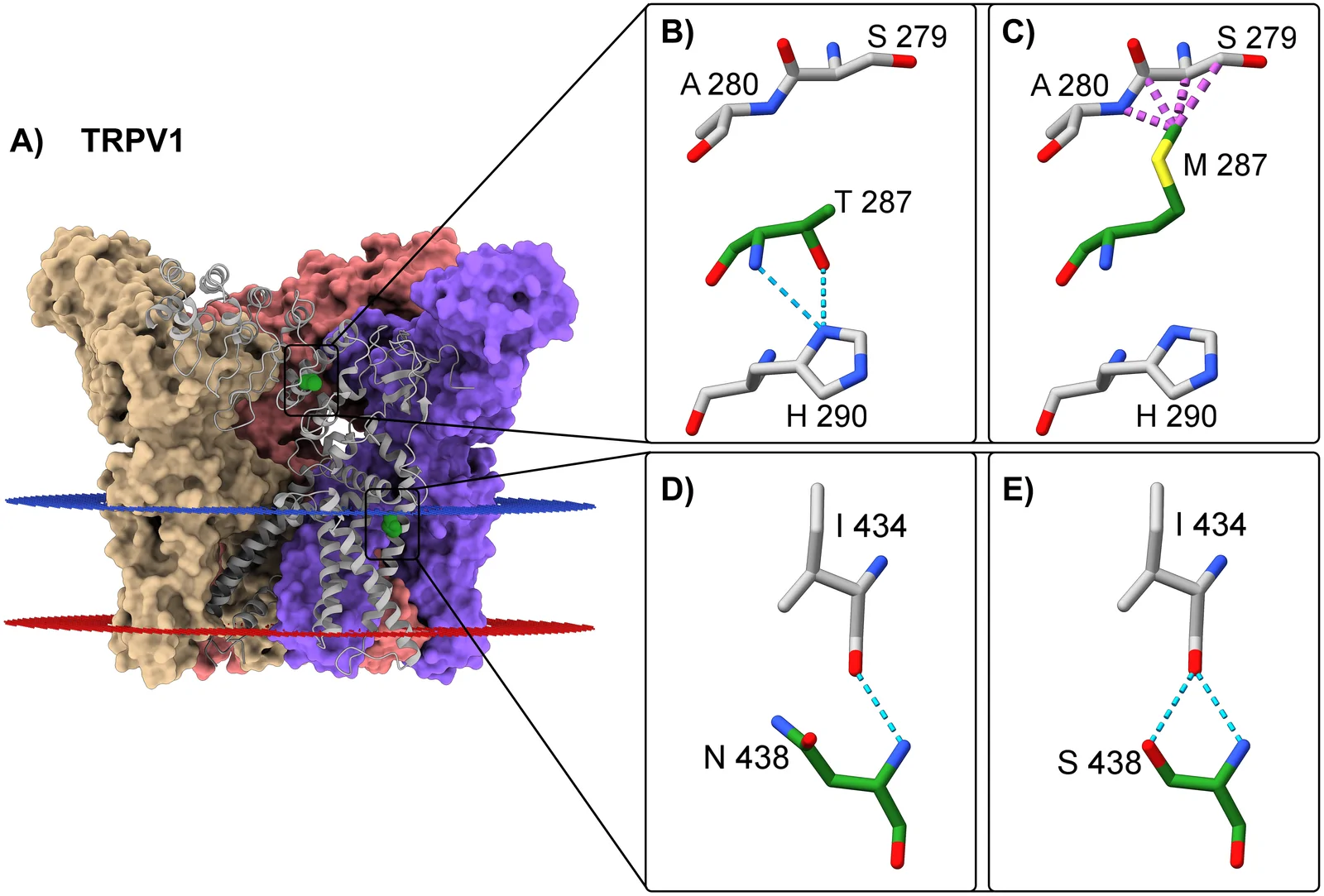
Analysis of *TRPV1* variants in the tetrameric ion channel structure. **(A)** Overall structure of *TRPV1* tetramer shown in cartoon and surface representation, with each monomer colored differently. Plasma membrane boundaries are indicated by red (extracellular) and blue (intracellular) lines. **(B)** Wild-type Thr287 forms hydrogen bonds (blue dashed lines) with His290. **(C)** Thr287Met substitution disrupts these hydrogen bonds and introduces steric clashes (purple dashed lines) with Ser279 and Ala280. **(D)** Wild-type Asn438 forms a hydrogen bond with Ile434 backbone. **(E)** Asn438Ser substitution maintains a similar hydrogen bonding pattern. A, Ala; H, His; I, Ile; M, Met; N, Asn; T, Thr; S, Ser.

*DAGLA* Ala750Val was predicted to be slightly stabilizing (DDMut ΔΔG = 0.14 kcal/mol) and had the lowest pathogenicity prediction among all variants (score 0.086) (**Figure 4A**). This variant lies within the C-terminal region predicted as intrinsically disordered by AIUPred (residues 684-1042), which also shows very low AlphaFold confidence (pLDDT = 38.2 at Ala750; mean 34.5 across residues 684–1042) (**Figure 4B**). The high surface accessibility (RSA = 0.82) and location in a flexible region (**Figure 4C**) are consistent with its likely benign classification (**Table 1**). *DAGLA* position 750 showed the most consistent tolerance profile: all 19 substitutions were neutral by FoldX (range -0.40 to +0.35 kcal/mol) and by DDMut, and all 19 were classified as likely benign by AlphaMissense, which places the position in the 9th percentile of mutational constraint across *DAGLA* by this predictor. Because the underlying coordinates lie in a region of very low model confidence (pLDDT 38.2), the FoldX and DDMut values computed here are treated as exploratory and are not interpretable in absolute terms, since agreement between two structure-dependent methods evaluated on unreliable coordinates does not provide independent support. The interpretation therefore rests mainly on evidence that does not depend on the specific low-confidence *DAGLA* coordinates used for the structure-based stability calculations, namely the AlphaMissense classification and the AIUPred disorder prediction.

**Figure 4:**
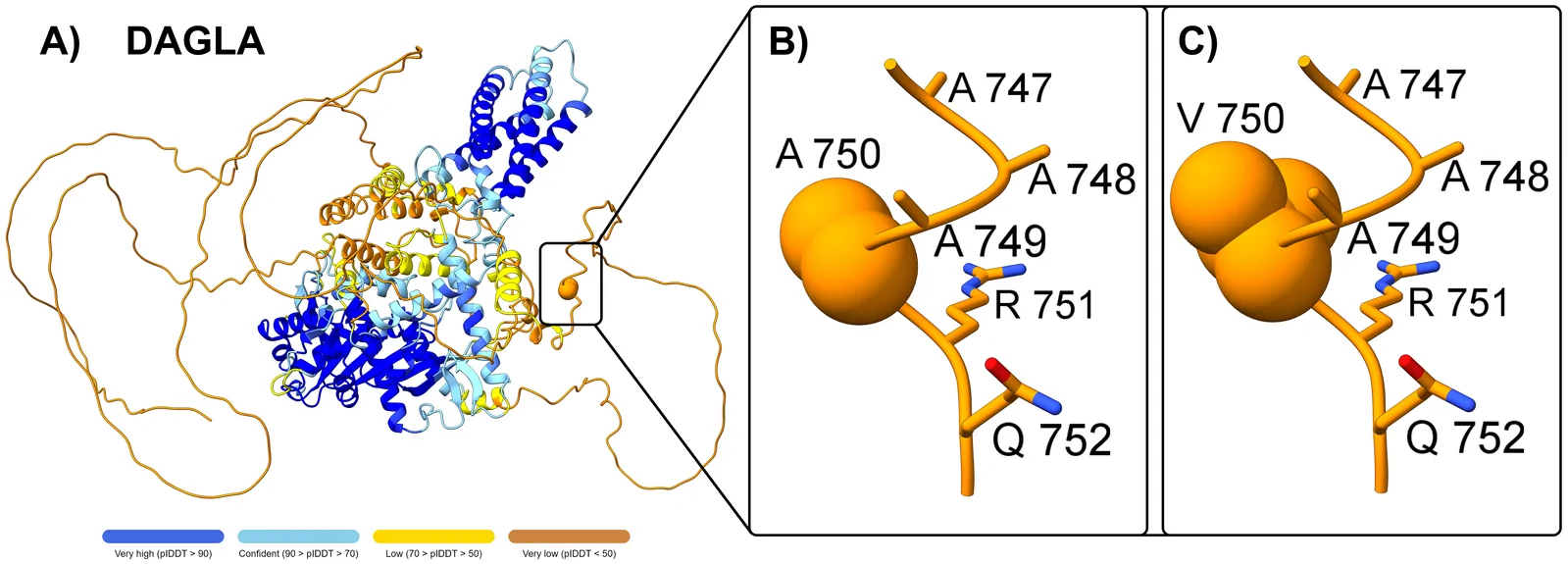
Structural context of the *DAGLA* p.(Ala750Val) variant in a disorder-predicted, low-confidence region. **(A)** Overall structure of *DAGLA* colored by AlphaFold pLDDT confidence scores: very high (blue, pLDDT > 90), confident (light blue, 70 < pLDDT < 90), low (yellow, 50 < pLDDT < 70), and very low (orange, pLDDT < 50). **(B)** Wild-type Ala750 shown in space-filling representation within the C-terminal region predicted as intrinsically disordered by AIUPred. **(C)** Modeled Ala750Val substitution within the same low-confidence region. A, Ala; R, Ar; Q, Gln; V, Val.

The eight positions differed markedly in mutational tolerance, from sites at which every substitution was destabilizing (*FAAH* Pro546, *NAAA* Leu325) to one at which none was (*DAGLA* Ala750). Agreement between methods was stronger at the level of the position than for the ranking of substitutions within a position, where it was weaker and, at *NAAA* Leu325 and *DAGLA* Ala750, absent (Spearman rho = -0.08 and -0.15 between FoldX and DDMut across the 19 substitutions). Notable discordances persisted even at the position level: at *TRPV1* Asn438 the two stability predictors indicate tolerance while AlphaMissense classifies 14 of 19 substitutions as likely pathogenic. On this basis, *FAAH* Arg326Pro showed the strongest and most concordant predicted impact; *FAAH* Pro546Leu and *NAAA* Leu325Phe lie at fragile positions but correspond to comparatively conservative substitutions, and their interpretation remains uncertain; and *DAGLA* position 750 showed a consistently tolerant profile across FoldX, DDMut and AlphaMissense, although the structure-dependent stability predictions at this site should be interpreted cautiously because of the very low AlphaFold confidence. These analyses are predictive and were used to interpret the structural context of each variant and to prioritize candidates for functional study.

### 3.4 Clinical features of the carriers of rare ECS variants

The carrier of the novel *FAAH* p.(Arg326Pro) variant was a 21-year-old woman at the time of blood collection, with severe obesity beginning in childhood. She weighed 110.8 kg and was 1.57 m tall, corresponding to a BMI of 45.0 kg/m². Waist, hip and neck circumferences were 135, 138 and 40 cm, respectively, with a waist-to-hip ratio of 0.98. Laboratory measurements included fasting plasma glucose of 100 mg/dL, total cholesterol of 198 mg/dL, HDL-C of 40 mg/dL, LDL-C of 97 mg/dL, triglycerides of 303 mg/dL, HbA1c of 5.1% and C-reactive protein of 0.51 mg/dL. The patient had diagnoses of metabolic syndrome and polycystic ovary syndrome.

Clinical characteristics of other patients with ECS variants are presented in **Table 2**.

## 4 Discussion

In this study, we identified rare variants in key genes of the ECS, including *FAAH*, *CNR1*, *NAAA*, *DAGLA*, *TRPV1*, and *NAPEPLD*, in a cohort of adults with severe obesity beginning before 21 years of age, predominantly during childhood. These variants were characterized by very low population frequencies, which is the criterion used to select them and does not in itself imply a functional or clinical impact (64b). Notably, the *FAAH* p.(Arg326Pro) variant was among those predicted to have a possible structural effect, although the *in silico* results were not fully consistent across prediction tools. Collectively, our findings expand the described spectrum of rare genetic variation in ECS genes in severe obesity.

Previous studies have focused on common polymorphisms within the ECS. For example, the functional *FAAH* c.385C>A p.(Pro129Thr) variant has been associated with increased adiposity across several cohorts, although results have been heterogeneous (79). In 2008, Monteleone et al. conducted a study on *FAAH* gene mutations, focusing on the c.385C>A variant in 115 individuals with obesity and binge eating disorder (BED), 74 individuals with obesity but without BED, and 110 controls. The study found a higher frequency of the C/A genotype and A allele in individuals with obesity, suggesting a potential link to obesity, though not specifically to BED (80).

This variability may reflect the complex interplay between the ECS and environmental modifiers. Of note, the identification of a loss-of-function *FAAH* variant p.(Arg315Ile) co-segregating with monogenic obesity in a family study (81) supports a possible causal link between *FAAH* dysfunction and childhood-onset obesity. In the present study no familial, segregation or functional data were available for p.(Arg326Pro); the variant is therefore reported as a rare finding of uncertain significance, and no causal inference is made.

Regarding the *CNR1*, common polymorphisms have been inconsistently associated with obesity and metabolic traits in diverse populations (5). The detection of rare *CNR1* variants in our cohort introduces a novel perspective. Functional studies would be required to determine whether the identified variants alter CB1 signaling in either direction, with divergent implications for energy homeostasis.

The role of the *CNR2* gene, which encodes CB2, in energy metabolism is not fully understood. However, studies have shown that variants in this gene may impact BMI, insulin resistance, and overall adiposity. For example, in a study involving 1,027 individuals with obesity, carriers of the A allele of the rs3123554 variant exhibited higher BMI, adiposity, waist circumference, insulin levels, HOMA-IR, and leptin levels compared to non-carriers of the A allele (82). Another study evaluated 82 patients with obesity before and after three months on a hypocaloric Mediterranean diet. The results indicated that non-carriers of the rs3123554 A allele experienced greater reductions in weight, BMI, waist circumference, blood pressure, total cholesterol, LDL, glucose, insulin, and HOMA-IR, suggesting a more favorable response to dietary treatment compared to A allele carriers (83).

Moreover, identifying rare variants in *NAPEPLD* and *NAAA*, enzymes that regulate endocannabinoid synthesis and degradation, provides original contributions to the field. A protective haplotype in *NAPEPLD* against severe obesity has been previously reported (84), suggesting that perturbations in endocannabinoid biosynthesis may influence susceptibility to weight gain. Likewise, alterations in *NAAA* could modulate palmitoylethanolamide levels, potentially impacting inflammation and metabolism (85). Rare variants were also identified in *TRPV1*, a receptor activated by anandamide, so that rare variation was found in several components of the pathway; whether it converges on a common phenotypic effect was not addressed here (86, 87).

Loss of *FAAH* function has been proposed to promote obesity by elevating endogenous anandamide levels, thereby enhancing CB1 receptor activation in hypothalamic circuits and peripheral tissues (37, 88). In animal models, *FAAH* inhibition or deletion increases adiposity, and this mechanism has been associated with hyperphagia, lipogenesis and energy storage. Whether the p.(Arg326Pro) substitution reduces *FAAH* activity, and therefore whether this mechanism applies to the carrier described here, was not tested in the present study and remains unknown.

Moreover, structural modeling provided hypothesis-generating mechanistic context for the identified variants. The *FAAH* p.(Arg326Pro) substitution, for instance, was predicted to be destabilizing by DDMut (ΔΔG = -1.11 kcal/mol), while structural modeling indicated loss of hydrogen bonds and a salt bridge within the amidase domain, at a site remote from the catalytic triad. The replacement of a flexible, polar arginine with a rigid, cyclic proline removes the side chain that donates the hydrogen bonds to Leu465 and Asp466 and, in the modeled rotamer, produces a steric overlap with Asp468. Whether such destabilization is sufficient to reduce anandamide hydrolysis *in vivo* cannot be determined from these data and would require enzymatic assays.

Similarly, the *NAAA* p.(Leu325Phe) variant, although predicted to have a lower pathogenicity score, was predicted to be destabilizing by DDMut (ΔΔG = -2.16 kcal/mol) within a buried hydrophobic core, suggesting potential impairment of protein folding or stability. Saturation analysis, however, showed that this position does not tolerate substitution in general and that phenylalanine is among its mildest replacements, so the magnitude of the ΔΔG value should not be read as evidence of a variant-specific effect. Overall, the structural predictions describe possible consequences at the protein level and generate hypotheses for functional testing.

*NAPEPLD* and *TRPV1* have established roles in energy balance: *NAPEPLD* generates anorexigenic lipids such as oleoylethanolamide, which contribute to satiety signaling (89, 90), and *TRPV1* participates in thermogenesis and in neuroendocrine satiety responses (87). Whether the variants identified here alter either function was not investigated in the present study.

The identification and characterization of rare genetic variants in severe obesity may contribute to etiological investigation and, when supported by robust clinical and functional evidence, to the development of precision-medicine approaches. Severe early-onset obesity, especially when accompanied by marked hyperphagia, is increasingly recognized as an indicator of underlying monogenic etiologies (91–94). Recent guidelines recommend genetic testing in such cases to improve diagnostic yield and enable tailored management (93). In this context, genes of the ECS, which have emerging roles in energy balance regulation, remain to be better characterized before they could be considered for inclusion in diagnostic panels alongside established obesity-related genes such as *LEP*, *LEPR*, *SH2B1*, *MC4R*, and *POMC* (8, 10, 55, 95, 96).

Understanding the genetic architecture of obesity presents significant opportunities for developing targeted interventions. Successful case studies in monogenic obesity illustrate how genetic diagnosis can inform therapeutic decisions. For example, congenital leptin deficiency is treatable with recombinant leptin therapy (97, 98), while defects in the melanocortin pathway can be addressed with *MC4R* agonists like setmelanotide, approved for specific genetic forms of obesity (99–101).

This dual role of the ECS in promoting fat storage and regulating energy expenditure underscores its potential as a therapeutic target for obesity and related metabolic disorders. Experimental studies of CB1 receptor antagonists have shown promising effects in animal models, including reductions in fat mass and improvements in insulin sensitivity, supporting ECS modulation as a potentially valuable metabolic intervention (30, 36, 102–104). Although no therapies are currently approved specifically for obesity associated with ECS gene variants, pharmacological CB1 receptor blockade has demonstrated clinical efficacy for weight loss; notably, rimonabant reduced body weight and improved cardiometabolic parameters before its withdrawal because of psychiatric adverse effects (36, 105). More recently, newer generations of peripherally restricted CB1 antagonists have been actively investigated to retain the metabolic benefits of CB1 blockade while minimizing central nervous system adverse effects (88, 106, 107).

Furthermore, monlunabant (MRI-1891, INV-202), an inverse agonist of the CB1 receptor, has gained prominence in recent years for decreasing food intake and weight in animals, as well as improving leptin sensitivity and glycemic parameters (108, 109). It is important to note that monlunabant is implicated in the inhibition of β-arrestin-2 recruitment driven by the CB1 receptor rather than the inhibition of adenylyl cyclase (109). In a phase 1b study, no serious or severe treatment-emergent adverse events were reported (110). Recently, Canadian individuals with obesity and metabolic syndrome participated in a placebo-controlled phase 2a clinical trial of monlunabant at varying doses (111). Knop and collaborators (2025) found a significant effect on weight loss; however, mild to moderate gastrointestinal and psychiatric adverse events appeared to be dose-related (111). Therefore, pharmacological therapy with the peripherally acting CB1R inverse agonist monlunabant seems promising, but requires further research to determine if it has an effective and clinically significant dose for the treatment of obesity.

In summary, rare variant screening in severe obesity may contribute to etiological investigation and genetic counseling, although the clinical utility of the ECS variants identified here remains uncertain. The present findings describe rare variation in ECS genes in this population; they do not demonstrate a contribution of these variants to the phenotype, and functional studies would be required before any clinical or therapeutic implication could be considered.

Several limitations should be considered. First, the small sample size limits the generalizability of the findings; with 12 carriers among 93 participants (12.9%; 95% CI 7.5–21.2%, Wilson method), the study was not powered to test enrichment, and no anthropometric or metabolic parameter differed significantly between carriers and non-carriers after correction for multiple testing (**Supplementary Table S2**). The absence of a significant difference does not establish equivalence between the two groups. Second, the pathogenicity of the identified variants was inferred from *in silico* predictions and structural modeling, without direct functional validation. Third, ΔΔG-based assessment has intrinsic limitations, as our data illustrate. The methods agreed more on which positions are constrained than on which substitution is most damaging within a position; within-position agreement between the two stability predictors was weak and, at two positions, absent. Agreement at the position level was also not universal, most clearly at *TRPV1* Asn438, where the stability predictors indicate tolerance and AlphaMissense does not. The AlphaFold model of *DAGLA* further has very low confidence in the region containing Ala750 (pLDDT = 38), so stability values computed there are not quantitatively interpretable. Fourth, obesity onset was recorded categorically rather than as an exact age, so the cohort cannot be stratified by age at onset. Therefore, while the data describe rare variation in ECS genes, no causal relationship with the phenotype can be established at this stage, and the computational results should be regarded as hypotheses requiring biological confirmation.

Given the preliminary nature of these findings, further studies are needed to validate the functional effects of the identified variants, particularly *FAAH* p.(Arg326Pro), through biochemical assays, expression analyses, and structural modeling. Establishing appropriate *in vivo* models may also clarify their impact on systemic energy regulation. Expanding cohort sizes and improving ancestral diversity will be important to better define the frequency and potential contribution of ECS-related variants to severe obesity with onset during childhood or adolescence.

## 5 Conclusion

Rare variants in key genes of the ECS, including *FAAH*, *CNR1*, *NAAA*, *DAGLA*, *TRPV1*, and *NAPEPLD*, were identified in adults with severe obesity, predominantly with childhood onset. Integrative structural and pathogenicity analyses prioritized the *FAAH* p.(Arg326Pro) substitution as the most likely to affect protein stability, through loss of a local polar interaction network within the amidase domain at a site distant from the catalytic triad. The remaining variants showed variable and, in some cases, discordant predictions across methods, and several of them correspond to substitutions that are among the better tolerated of the 19 possible at their own position. *DAGLA* position 750 showed the most consistent predicted tolerance, with all substitutions classified as neutral by FoldX and DDMut and as likely benign by AlphaMissense.

These findings describe rare variation in ECS components in this cohort. The pathogenic significance of these variants remains to be established: the structural and saturation analyses presented here are predictive and provide computational evidence for prioritizing candidates such as *FAAH* p.(Arg326Pro), but do not by themselves demonstrate a contribution to the phenotype. Further studies involving biochemical assays, *in vivo* models, and expanded clinical cohorts are necessary to clarify the biological effects of ECS-related variants and to determine their potential relevance for precision approaches to the diagnosis and management of severe obesity.

## Data Availability

The datasets presented in this study can be found in online repositories. The names of the repository/repositories and accession number(s) can be found in the article/**Supplementary Material**.
