# Supplementary material for: Rare endocannabinoid system variants identified by whole-exome sequencing and structural modeling in adults with severe obesity predominantly of childhood onset
Source: Front Endocrinol (Lausanne). 2026 Sep 25;17:1862926. doi: 10.3389/fendo.2026.1862926 (PMC13649445; doi:10.3389/fendo.2026.1862926)
Supplement: Supplementary file 1 [file DataSheet1.pdf]

Supplementary Material

Contents

Supplementary Table S1. Mutational profile of the eight variant positions
Supplementary Table S2. Anthropometric and metabolic comparison of carriers versus non-carriers
Supplementary Figure S1. In silico saturation mutagenesis at the eight variant positions
Supplementary Dataset. Complete saturation-mutagenesis results (152 substitutions)

Supplementary Table S1. Mutational profile of the eight variant positions

Supplementary Table S1. For each variant position, all 19 possible amino-acid substitutions were evaluated with FoldX, Pythia, DDMut and AlphaMissense (AM). The table reports, per tool, the median effect at the position, the number of substitutions in each class, the mutational-constraint percentile of the position relative to all positions of the same protein, and the value obtained for the substitution observed in the patient.

Position	Observed substitution	FoldX median ΔΔG	FoldX N/D/HD	FoldX ΔΔG (obs.)	DDMut median ΔΔG	DDMut ΔΔG (obs.)	Pythia %ile	AM LP/Amb/LB	AM %ile	AM score (obs.)
FAAH Arg326	Arg326Pro	1.62	0/10/9	3.04	-0.24	-1.11	58	7/5/7	55	0.8837 (LPath)
FAAH Pro546	Pro546Leu	3.94	0/0/19	3.43	-1.94	-1.68	96	15/4/0	81	0.5375 (Amb)
CNR1 Ile227	Ile227Val	0.56	6/10/3	0.49	-0.31	-0.12	86	15/1/3	68	0.2105 (LBen)
NAAA Leu325	Leu325Phe	4.48	0/2/17	2.56	-1.82	-2.16	94	13/2/4	72	0.2247 (LBen)
DAGLA Ala750	Ala750Val	-0.06	19/0/0	0.32	0.08	0.14	59	0/0/19	9	0.0855 (LBen)
TRPV1 Thr287	Thr287Met	1.92	2/8/9	1.83	-0.96	-0.26	59	13/3/3	54	0.1609 (LBen)
TRPV1 Asn438	Asn438Ser	-0.06	14/4/1	0.29	0.02	0.08	39	14/3/2	58	0.1317 (LBen)
NAPEPLD Arg153	Arg153His	1.4	5/9/5	3.31	-0.78	-0.2	15	13/1/5	53	0.1651 (LBen)

ΔΔG values are in kcal/mol for FoldX and DDMut (positive = destabilizing for FoldX; negative = destabilizing for DDMut). Pythia returns a relative score proportional to ΔΔG (log-probability ratio), not calibrated in kcal/mol. FoldX N/D/HD = number of the 19 substitutions that are neutral-or-stabilizing / destabilizing / highly destabilizing. AM LP/Amb/LB = number classified by AlphaMissense as likely pathogenic / ambiguous / likely benign. %ile = percentile of positional mutational constraint within the protein.

Supplementary Table S2. Anthropometric and metabolic comparison of carriers versus non-carriers

Supplementary Table S2. Carriers of rare ECS variants were compared with non-carriers across sixteen anthropometric and metabolic parameters using the two-sided Mann–Whitney U test. p = uncorrected p-value; q (BH) = value after Benjamini–Hochberg correction at a false discovery rate of 5%. No parameter differed significantly after correction. Missing values were not imputed; group sizes reflect participants with an available measurement for each parameter.

Parameter	n (carriers)	n (non-carriers)	Carriers, median (Q1; Q3)	Non-carriers, median (Q1; Q3)	p	q (BH)
Age (years)	12	82	42.5 (34.5; 48.0)	37.5 (28.0; 46.5)	0.472	0.835
Weight (kg)	12	82	123.5 (104.1; 145.1)	136.2 (120.9; 160.8)	0.059	0.316
BMI (kg/m2)	12	82	46.4 (44.5; 49.9)	50.0 (45.5; 57.6)	0.111	0.446
Waist circumference (cm)	12	82	136.3 (126.3; 143.5)	138.8 (128.8; 153.0)	0.310	0.828
Hip circumference (cm)	12	82	137.0 (133.0; 147.9)	147.0 (136.0; 157.2)	0.047	0.316
Waist-to-hip ratio	12	82	1.0 (0.9; 1.0)	1.0 (0.9; 1.0)	0.522	0.835
Neck circumference (cm)	12	71	42.8 (40.0; 45.9)	43.0 (40.0; 46.0)	0.751	0.858
Total cholesterol (mg/dL)	11	63	198.0 (153.0; 227.0)	190.0 (166.0; 225.0)	0.638	0.858
LDL cholesterol (mg/dL)	10	62	122.7 (96.0; 151.7)	117.5 (96.6; 144.3)	0.961	0.961
HDL cholesterol (mg/dL)	11	63	43.0 (40.0; 52.0)	46.0 (41.0; 53.0)	0.704	0.858
Triglycerides (mg/dL)	11	63	121.0 (105.0; 179.0)	116.0 (77.0; 155.0)	0.281	0.828
Fasting glucose (mg/dL)	8	46	97.9 (87.3; 124.0)	97.5 (89.8; 105.3)	0.874	0.932
HbA1c (%)	9	46	6.2 (4.9; 6.7)	5.8 (5.5; 6.3)	0.665	0.858
C-reactive protein (mg/dL)	9	46	1.3 (0.6; 2.0)	1.5 (0.6; 3.2)	0.495	0.835
Leptin (pg/mL)	12	81	1833.1 (99.0; 3231.3)	2408.3 (1131.0; 3303.3)	0.365	0.834
Adiponectin (ng/mL)	8	58	2976.7 (1618.7; 7573.8)	1542.0 (862.9; 2327.2)	0.047	0.316

Note: the non-carrier group size shown here is being reconciled with the total participant count (n = 93 in the main text); the corresponding p- and q-values will be confirmed accordingly before final submission.

Supplementary Figure S1. In silico saturation mutagenesis at the eight variant positions

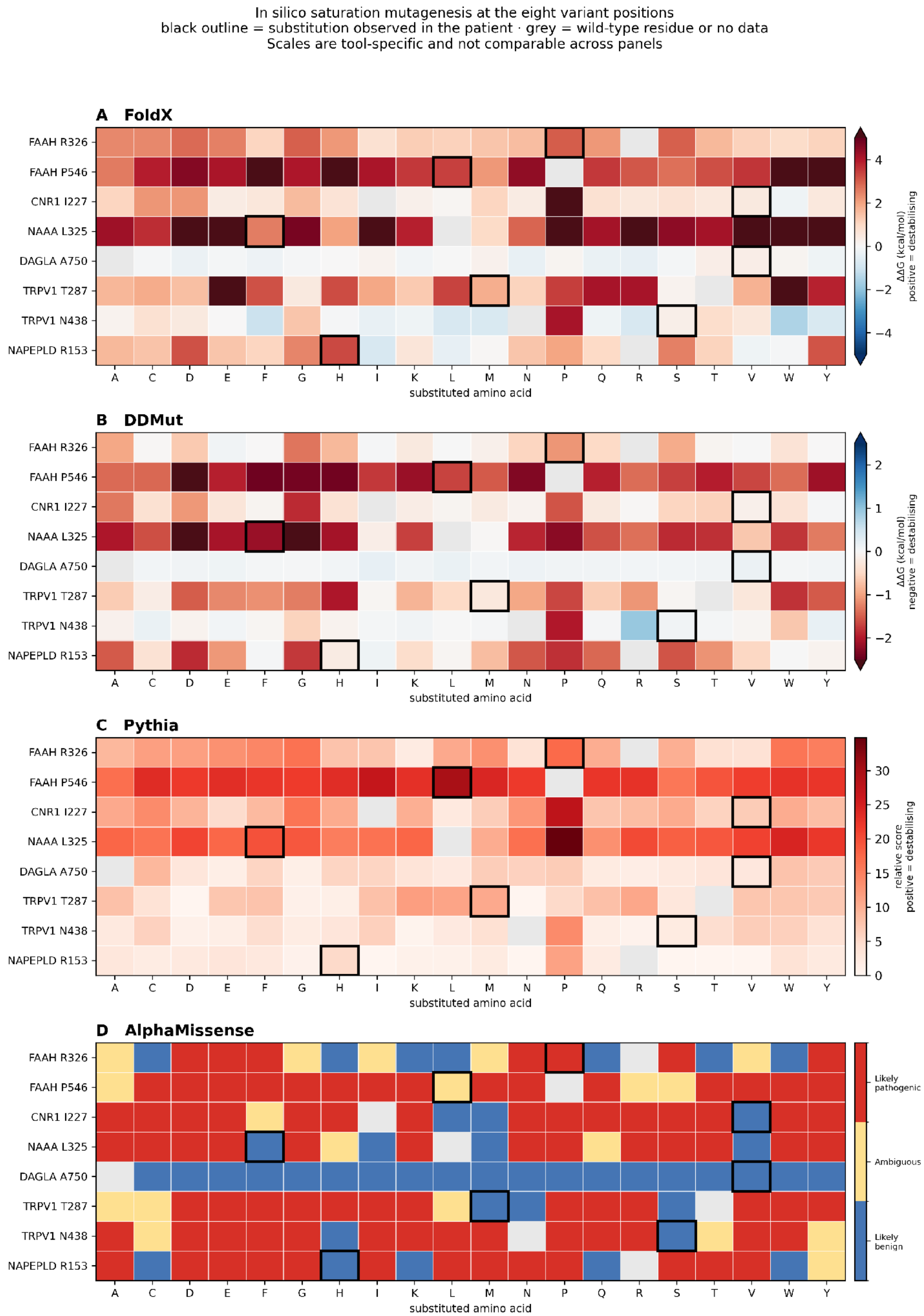

Supplementary Figure S1. Heat maps of in silico saturation mutagenesis at the eight variant positions. Each panel corresponds to one method: (A) FoldX ΔΔG, (B) DDMut ΔΔG, (C) Pythia relative score, and (D) AlphaMissense class. Rows are the eight variant positions and columns the 20 amino acids. Black outlines mark the substitution observed in the patient; grey cells indicate the wild-type residue. Scales are tool-specific and are not comparable across panels.

Supplementary Dataset. Complete saturation-mutagenesis results (152 substitutions)

Supplementary Dataset. Complete per-substitution results underlying Supplementary Table S2 and Supplementary Figure S1: all 19 possible substitutions at each of the eight variant positions (152 in total), scored by FoldX (mean ± SD of five runs), Pythia, DDMut and AlphaMissense. Rows corresponding to the substitution observed in a patient are shown in bold and flagged in the last column.

Position	Substitution	FoldX ΔΔG ± SD	Pythia	DDMut ΔΔG	AlphaMissense	AM class	Observed
FAAH Arg326	Arg326Ala	2.41 ± 0.05	9.03	-0.97	0.5422	Amb	
FAAH Arg326	Arg326Cys	2.40 ± 0.05	11.59	-0.03	0.1419	LBen	
FAAH Arg326	Arg326Asp	2.85 ± 0.01	11.70	-0.66	0.8915	LPath	
FAAH Arg326	Arg326Glu	2.53 ± 0.04	13.15	0.08	0.6105	LPath	
FAAH Arg326	Arg326Phe	1.06 ± 0.15	14.39	-0.01	0.7222	LPath	
FAAH Arg326	Arg326Gly	2.99 ± 0.05	16.59	-1.33	0.5423	Amb	
FAAH Arg326	Arg326His	2.18 ± 0.04	7.74	-0.81	0.1369	LBen	
FAAH Arg326	Arg326Ile	0.78 ± 0.04	7.55	0.02	0.3592	Amb	
FAAH Arg326	Arg326Lys	1.23 ± 0.05	2.03	-0.24	0.1437	LBen	
FAAH Arg326	Arg326Leu	1.17 ± 0.38	10.50	-0.0	0.3065	LBen	
FAAH Arg326	Arg326Met	1.39 ± 0.09	13.30	-0.13	0.5001	Amb	
FAAH Arg326	Arg326Asn	1.54 ± 0.21	3.75	-0.53	0.7744	LPath	
***FAAH* Arg326**	**Arg326Pro**	**3.04 ± 0.07**	**17.37**	**-1.11**	**0.8837**	**LPath**	**Yes**
FAAH Arg326	Arg326Gln	2.15 ± 0.33	10.15	-0.49	0.1418	LBen	
FAAH Arg326	Arg326Ser	2.98 ± 0.04	9.96	-0.93	0.6483	LPath	
FAAH Arg326	Arg326Thr	1.62 ± 0.28	4.21	-0.07	0.2988	LBen	
FAAH Arg326	Arg326Val	1.15 ± 0.05	3.82	0.0	0.3596	Amb	
FAAH Arg326	Arg326Trp	0.92 ± 0.09	16.10	-0.35	0.2816	LBen	
FAAH Arg326	Arg326Tyr	1.13 ± 0.12	15.12	-0.01	0.5706	LPath	
FAAH Pro546	Pro546Ala	2.60 ± 0.01	16.80	-1.41	0.3925	Amb	
FAAH Pro546	Pro546Cys	3.94 ± 0.46	23.58	-1.44	0.8343	LPath	
FAAH Pro546	Pro546Asp	4.59 ± 0.13	21.95	-2.72	0.9105	LPath	
FAAH Pro546	Pro546Glu	4.09 ± 0.02	23.32	-1.94	0.8261	LPath	
FAAH Pro546	Pro546Phe	5.37 ± 0.27	22.40	-2.44	0.9263	LPath	
FAAH Pro546	Pro546Gly	4.05 ± 0.01	21.84	-2.39	0.7604	LPath	
FAAH Pro546	Pro546His	6.68 ± 2.76	23.47	-2.42	0.6002	LPath	
FAAH Pro546	Pro546Ile	4.08 ± 0.46	26.82	-1.77	0.9152	LPath	
FAAH Pro546	Pro546Lys	3.54 ± 0.58	23.07	-2.15	0.7647	LPath	
***FAAH* Pro546**	**Pro546Leu**	**3.43 ± 0.31**	**29.80**	**-1.68**	**0.5375**	**Amb**	**Yes**
FAAH Pro546	Pro546Met	2.17 ± 0.25	24.29	-1.53	0.8787	LPath	
FAAH Pro546	Pro546Asn	4.45 ± 0.14	21.93	-2.32	0.8464	LPath	

Position	Substitution	FoldX ΔΔG ± SD	Pythia	DDMut ΔΔG	AlphaMissense	AM class	Observed
FAAH Pro546	Pro546Gln	3.54 ± 0.10	22.39	-1.94	0.5965	LPath	
FAAH Pro546	Pro546Arg	3.10 ± 0.56	22.85	-1.4	0.4865	Amb	
FAAH Pro546	Pro546Ser	2.74 ± 0.03	15.92	-1.71	0.5062	Amb	
FAAH Pro546	Pro546Thr	3.24 ± 0.22	19.43	-1.96	0.5644	LPath	
FAAH Pro546	Pro546Val	3.58 ± 0.08	21.64	-1.67	0.7881	LPath	
FAAH Pro546	Pro546Trp	6.18 ± 2.11	22.86	-1.4	0.9073	LPath	
FAAH Pro546	Pro546Tyr	6.02 ± 1.39	22.40	-2.12	0.887	LPath	
CNR1 Ile227	Ile227Ala	1.06 ± 0.00	10.59	-1.32	0.859	LPath	
CNR1 Ile227	Ile227Cys	2.21 ± 0.03	13.81	-0.38	0.9691	LPath	
CNR1 Ile227	Ile227Asp	2.21 ± 0.01	9.47	-1.08	0.9898	LPath	
CNR1 Ile227	Ile227Glu	0.41 ± 0.03	4.86	-0.31	0.968	LPath	
CNR1 Ile227	Ile227Phe	0.56 ± 0.01	8.78	-0.06	0.5103	Amb	
CNR1 Ile227	Ile227Gly	1.57 ± 0.04	16.24	-1.87	0.9847	LPath	
CNR1 Ile227	Ile227His	0.67 ± 0.02	10.38	-0.37	0.9364	LPath	
CNR1 Ile227	Ile227Lys	0.27 ± 0.02	9.94	-0.19	0.9187	LPath	
CNR1 Ile227	Ile227Leu	0.10 ± 0.00	2.54	-0.07	0.2461	LBen	
CNR1 Ile227	Ile227Met	1.09 ± 0.01	6.66	-0.23	0.2963	LBen	
CNR1 Ile227	Ile227Asn	0.42 ± 0.01	12.65	-0.08	0.939	LPath	
CNR1 Ile227	Ile227Pro	5.62 ± 0.08	27.60	-1.57	0.9949	LPath	
CNR1 Ile227	Ile227Gln	0.76 ± 0.06	7.49	-0.24	0.9406	LPath	
CNR1 Ile227	Ile227Arg	0.56 ± 0.01	8.44	-0.02	0.8744	LPath	
CNR1 Ile227	Ile227Ser	0.73 ± 0.00	10.48	-0.53	0.9269	LPath	
CNR1 Ile227	Ile227Thr	0.54 ± 0.01	5.95	-0.57	0.7535	LPath	
***CNR1* Ile227**	**Ile227Val**	**0.49 ± 0.00**	**6.68**	**-0.12**	**0.2105**	**LBen**	**Yes**
CNR1 Ile227	Ile227Trp	-0.20 ± 0.01	10.28	-0.35	0.9152	LPath	
CNR1 Ile227	Ile227Tyr	0.54 ± 0.00	8.30	0.02	0.8463	LPath	
NAAA Leu325	Leu325Ala	4.23 ± 0.01	17.77	-2.03	0.646	LPath	
NAAA Leu325	Leu325Cys	3.71 ± 0.01	16.35	-1.61	0.7211	LPath	
NAAA Leu325	Leu325Asp	5.72 ± 0.01	20.93	-2.53	0.9353	LPath	
NAAA Leu325	Leu325Glu	5.52 ± 0.03	17.57	-2.07	0.8096	LPath	
***NAAA* Leu325**	**Leu325Phe**	**2.56 ± 0.05**	**19.67**	**-2.16**	**0.2247**	**LBen**	**Yes**
NAAA Leu325	Leu325Gly	4.73 ± 0.02	19.18	-2.72	0.8238	LPath	
NAAA Leu325	Leu325His	2.00 ± 0.06	15.37	-2.08	0.5626	Amb	
NAAA Leu325	Leu325Ile	6.14 ± 0.44	16.47	-0.17	0.2469	LBen	

Position	Substitution	FoldX ΔΔG ± SD	Pythia	DDMut ΔΔG	AlphaMissense	AM class	Observed
NAAA Leu325	Leu325Lys	3.88 ± 0.59	17.80	-1.7	0.7829	LPath	
NAAA Leu325	Leu325Met	0.96 ± 0.09	10.25	-0.02	0.2178	LBen	
NAAA Leu325	Leu325Asn	2.92 ± 0.00	16.70	-1.9	0.7699	LPath	
NAAA Leu325	Leu325Pro	8.09 ± 0.04	34.84	-2.26	0.7347	LPath	
NAAA Leu325	Leu325Gln	4.16 ± 0.04	13.22	-1.6	0.5114	Amb	
NAAA Leu325	Leu325Arg	9.59 ± 1.04	20.28	-1.37	0.6163	LPath	
NAAA Leu325	Leu325Ser	4.48 ± 0.01	18.32	-1.87	0.6396	LPath	
NAAA Leu325	Leu325Thr	4.15 ± 0.04	19.06	-1.82	0.5925	LPath	
NAAA Leu325	Leu325Val	5.77 ± 0.02	21.23	-0.68	0.2846	LBen	
NAAA Leu325	Leu325Trp	11.45 ± 0.56	24.57	-1.78	0.5968	LPath	
NAAA Leu325	Leu325Tyr	7.76 ± 0.06	22.21	-1.29	0.7366	LPath	
DAGLA Ala750	Ala750Cys	-0.06 ± 0.00	9.05	0.07	0.2115	LBen	
DAGLA Ala750	Ala750Asp	-0.30 ± 0.00	3.06	0.09	0.1214	LBen	
DAGLA Ala750	Ala750Glu	-0.08 ± 0.02	1.47	0.1	0.109	LBen	
DAGLA Ala750	Ala750Phe	-0.25 ± 0.04	5.68	0.04	0.1888	LBen	
DAGLA Ala750	Ala750Gly	-0.06 ± 0.00	1.33	0.03	0.0712	LBen	
DAGLA Ala750	Ala750His	0.03 ± 0.02	5.58	0.07	0.1846	LBen	
DAGLA Ala750	Ala750Ile	0.16 ± 0.03	5.58	0.21	0.1465	LBen	
DAGLA Ala750	Ala750Lys	-0.14 ± 0.04	3.47	0.08	0.1768	LBen	
DAGLA Ala750	Ala750Leu	-0.02 ± 0.03	2.34	0.17	0.1007	LBen	
DAGLA Ala750	Ala750Met	0.20 ± 0.00	6.61	0.07	0.1568	LBen	
DAGLA Ala750	Ala750Asn	-0.38 ± 0.03	6.04	0.12	0.1268	LBen	
DAGLA Ala750	Ala750Pro	0.12 ± 0.04	6.67	0.09	0.0737	LBen	
DAGLA Ala750	Ala750Gln	-0.35 ± 0.00	2.07	0.08	0.1212	LBen	
DAGLA Ala750	Ala750Arg	-0.40 ± 0.01	1.58	0.07	0.1429	LBen	
DAGLA Ala750	Ala750Ser	-0.10 ± 0.00	2.52	0.07	0.0716	LBen	
DAGLA Ala750	Ala750Thr	0.35 ± 0.00	3.21	0.08	0.0685	LBen	
***DAGLA* Ala750**	**Ala750Val**	**0.32 ± 0.00**	**2.78**	**0.14**	**0.0855**	**LBen**	**Yes**
DAGLA Ala750	Ala750Trp	0.15 ± 0.05	7.65	0.05	0.3309	LBen	
DAGLA Ala750	Ala750Tyr	-0.21 ± 0.03	6.88	0.07	0.2372	LBen	
TRPV1 Thr287	Thr287Ala	1.66 ± 0.00	7.96	-0.63	0.5002	Amb	
TRPV1 Thr287	Thr287Cys	1.85 ± 0.01	3.81	-0.18	0.5392	Amb	
TRPV1 Thr287	Thr287Asp	1.51 ± 1.09	-0.33	-1.52	0.8841	LPath	
TRPV1 Thr287	Thr287Glu	5.54 ± 1.19	7.47	-1.21	0.934	LPath	

Position	Substitution	FoldX ΔΔG ± SD	Pythia	DDMut ΔΔG	AlphaMissense	AM class	Observed
TRPV1 Thr287	Thr287Phe	3.18 ± 1.48	7.70	-1.15	0.8023	LPath	
TRPV1 Thr287	Thr287Gly	0.50 ± 0.00	1.08	-1.27	0.6804	LPath	
TRPV1 Thr287	Thr287His	3.26 ± 0.10	1.32	-2.02	0.6256	LPath	
TRPV1 Thr287	Thr287Ile	1.92 ± 1.91	7.99	-0.05	0.8137	LPath	
TRPV1 Thr287	Thr287Lys	1.30 ± 0.26	11.72	-0.84	0.9029	LPath	
TRPV1 Thr287	Thr287Leu	3.37 ± 2.62	11.13	-0.49	0.3525	Amb	
***TRPV1* Thr287**	**Thr287Met**	**1.83 ± 0.22**	**10.57**	**-0.26**	**0.1609**	**LBen**	**Yes**
TRPV1 Thr287	Thr287Asn	1.12 ± 0.08	-1.50	-0.96	0.2208	LBen	
TRPV1 Thr287	Thr287Pro	3.46 ± 0.90	5.11	-1.67	0.8914	LPath	
TRPV1 Thr287	Thr287Gln	4.13 ± 0.37	8.13	-0.62	0.7663	LPath	
TRPV1 Thr287	Thr287Arg	4.13 ± 2.67	10.76	-1.11	0.7984	LPath	
TRPV1 Thr287	Thr287Ser	0.18 ± 0.01	2.89	-0.04	0.2352	LBen	
TRPV1 Thr287	Thr287Val	1.72 ± 0.37	7.41	-0.29	0.629	LPath	
TRPV1 Thr287	Thr287Trp	12.12 ± 0.96	7.40	-1.8	0.9502	LPath	
TRPV1 Thr287	Thr287Tyr	3.84 ± 0.98	7.06	-1.53	0.805	LPath	
TRPV1 Asn438	Asn438Ala	0.17 ± 0.02	2.56	-0.11	0.7817	LPath	
TRPV1 Asn438	Asn438Cys	0.75 ± 0.01	6.09	0.16	0.4787	Amb	
TRPV1 Asn438	Asn438Asp	0.48 ± 0.03	1.00	-0.08	0.6481	LPath	
TRPV1 Asn438	Asn438Glu	-0.00 ± 0.04	1.45	0.06	0.9443	LPath	
TRPV1 Asn438	Asn438Phe	-1.07 ± 0.03	4.59	-0.04	0.9206	LPath	
TRPV1 Asn438	Asn438Gly	1.38 ± 0.02	1.83	-0.56	0.6674	LPath	
TRPV1 Asn438	Asn438His	-0.06 ± 0.05	3.26	-0.08	0.2188	LBen	
TRPV1 Asn438	Asn438Ile	-0.37 ± 0.07	6.18	0.05	0.8778	LPath	
TRPV1 Asn438	Asn438Lys	-0.21 ± 0.04	0.53	0.08	0.9002	LPath	
TRPV1 Asn438	Asn438Leu	-0.80 ± 0.06	2.32	0.01	0.7497	LPath	
TRPV1 Asn438	Asn438Met	-0.75 ± 0.03	4.17	0.02	0.8771	LPath	
TRPV1 Asn438	Asn438Pro	4.12 ± 0.19	13.84	-2.0	0.9439	LPath	
TRPV1 Asn438	Asn438Gln	-0.23 ± 0.01	1.37	0.02	0.7903	LPath	
TRPV1 Asn438	Asn438Arg	-0.81 ± 0.06	0.59	0.94	0.8454	LPath	
***TRPV1* Asn438**	**Asn438Ser**	**0.29 ± 0.01**	**2.17**	**0.08**	**0.1317**	**LBen**	**Yes**
TRPV1 Asn438	Asn438Thr	0.87 ± 0.01	4.51	0.05	0.5305	Amb	
TRPV1 Asn438	Asn438Val	0.56 ± 0.07	6.54	-0.05	0.8353	LPath	
TRPV1 Asn438	Asn438Trp	-1.54 ± 0.03	6.25	-0.67	0.9186	LPath	
TRPV1 Asn438	Asn438Tyr	-0.75 ± 0.04	3.92	0.18	0.5102	Amb	

Position	Substitution	FoldX ΔΔG ± SD	Pythia	DDMut ΔΔG	AlphaMissense	AM class	Observed
NAPEPLD Arg153	Arg153Ala	1.62 ± 0.11	2.96	-1.54	0.8105	LPath	
NAPEPLD Arg153	Arg153Cys	1.42 ± 0.10	1.80	-0.36	0.2027	LBen	
NAPEPLD Arg153	Arg153Asp	3.17 ± 0.21	2.52	-1.86	0.9622	LPath	
NAPEPLD Arg153	Arg153Glu	1.40 ± 0.36	3.15	-1.09	0.8133	LPath	
NAPEPLD Arg153	Arg153Phe	1.06 ± 0.32	2.62	0.02	0.6547	LPath	
NAPEPLD Arg153	Arg153Gly	2.43 ± 0.11	0.70	-1.76	0.7235	LPath	
***NAPEPLD* Arg153**	**Arg153His**	**3.31 ± 0.45**	**5.12**	**-0.2**	**0.1651**	**LBen**	**Yes**
NAPEPLD Arg153	Arg153Ile	-0.82 ± 0.06	-4.15	0.11	0.6948	LPath	
NAPEPLD Arg153	Arg153Lys	0.60 ± 0.19	0.89	-0.44	0.2837	LBen	
NAPEPLD Arg153	Arg153Leu	-0.41 ± 0.05	0.93	-0.05	0.6763	LPath	
NAPEPLD Arg153	Arg153Met	0.07 ± 0.06	2.15	-0.78	0.7231	LPath	
NAPEPLD Arg153	Arg153Asn	1.40 ± 0.20	-1.64	-1.55	0.8996	LPath	
NAPEPLD Arg153	Arg153Pro	1.98 ± 0.09	11.38	-1.83	0.9739	LPath	
NAPEPLD Arg153	Arg153Gln	1.01 ± 0.32	1.91	-1.43	0.2372	LBen	
NAPEPLD Arg153	Arg153Ser	2.51 ± 0.09	-0.66	-1.57	0.8352	LPath	
NAPEPLD Arg153	Arg153Thr	1.13 ± 0.22	-3.83	-1.12	0.6399	LPath	
NAPEPLD Arg153	Arg153Val	-0.23 ± 0.08	-3.16	-0.52	0.6559	LPath	
NAPEPLD Arg153	Arg153Trp	-0.06 ± 0.43	3.77	0.0	0.2302	LBen	
NAPEPLD Arg153	Arg153Tyr	3.14 ± 1.04	2.20	-0.09	0.4188	Amb	

ΔΔG in kcal/mol (FoldX, DDMut). Pythia is a relative score (not kcal/mol). AlphaMissense score ranges 0–1; AM class: LPath = likely pathogenic, Amb = ambiguous, LBen = likely benign.
